# Preclinical evaluation of exemestane as a novel chemotherapy for gastric cancer

**DOI:** 10.1111/jcmm.14605

**Published:** 2019-09-26

**Authors:** Juan‐Cheng Yang, Ning Chang, Deng‐Chyang Wu, Wei‐Chung Cheng, Wei‐Min Chung, Wei‐Chun Chang, Fu‐Ju Lei, Chung‐Jung Liu, I‐Chen Wu, Hsueh‐Chou Lai, Wen‐Lung Ma

**Affiliations:** ^1^ Department of Gastroenterology, Chinese Medicine Research and Development Center, Sex Hormone Research Center, Research Center for Tumor Medicine China Medical University Hospital Taichung Taiwan; ^2^ Department of OBS & GYN China Medical University Hospital Taichung Taiwan; ^3^ Graduate Institute of Chinese Medicine, Graduate Institute of BioMedical Sciences, School of Medicine China Medical University Taichung Taiwan; ^4^ Department of Medicine China Medical University Taichung Taiwan; ^5^ Department of Medicine Center for Stem Cell Research Kaohsiung Medical University Kaohsiung Taiwan; ^6^ Division of Gastroenterology, Department of Internal Medicine Kaohsiung Medical University Hospital Kaohsiung Taiwan; ^7^ Department of Nursing Asia University Taichung Taiwan

**Keywords:** aromatase, exemestane, gastric cancer

## Abstract

CYP19A1/aromatase (Ar) is a prognostic biomarker of gastric cancer (GCa). Ar is a critical enzyme for converting androstenedione to oestradiol in the steroidogenesis cascade. For decades, Ar has been targeted with Ar inhibitors (ARIs) in gynaecologic malignancies; however, it is unexplored in GCa. A single‐cohort tissue microarray examination was conducted to study the association between Ar expression and disease outcome in Asian patients with GCa. The results revealed that Ar was a prognostic promoter. Bioinformatics analyses conducted on a Caucasian‐based cDNA microarray databank showed Ar to be positively associated with GCa prognosis for multiple clinical modalities, including surgery, 5‐Fluorouracil (5‐FU) for adjuvant chemotherapy, or HER2 positivity. These findings imply that targeting Ar expression exhibits a potential for fulfilling unmet medical needs. Hence, Ar‐targeting compounds were tested, and the results showed that exemestane exhibited superior cancer‐suppressing efficacy to other ARIs. In addition, exemestane down‐regulated Ar expression. Ablating Ar abundance with short hairpin (sh)Ar could also suppress GCa cell growth, and adding 5‐FU could facilitate this effect. Notably, adding oestradiol could not prevent exemestane or shAr effects, implicating a nonenzymatic mechanism of Ar in cancer growth. Regarding translational research, treatment with exemestane alone exhibited tumour suppression efficacy in a dose‐dependent manner. Combining subminimal doses of 5‐FU and exemestane exerted an excellent tumour suppression effect without influencing bodyweight. This study validated the therapeutic potentials of exemestane in GCa. Combination of metronomic 5‐FU and exemestane for GCa therapy is recommended.

## 1 INTRODUCTION

Gastric cancer (GCa) is the third leading cause of cancer‐related mortality in the world.^1^ The incidence of GCa has been reported to vary worldwide,^2^, ^3^ and GCa has a poor prognosis with an only ≤10% 5‐year survival rate.^4^ Most patients are diagnosed at an advanced stage, or they rapidly experience relapse within 12 months after surgery.^4^, ^5^, ^6^ Resection is the first‐of‐choice treatment modality; nevertheless, it is associated with a high recurrence rate.^6^ Conversely, chemotherapy is often effective in patients with early‐stage GCa; however, poor prognosis is still presented in patients with advanced GCa.^2^, ^4^, ^7^ Therefore, there is high clinical demand for new adjuvant chemotherapy for GCa.^2^

Gastrectomy is the main therapeutic modality for GCa. Nevertheless, postsurgical recurrence is often observed in advanced disease.^8^ Therefore, adjuvant chemotherapy is used for secondary prevention.^4^ Among various chemoagents, 5‐Fluorouracil (5‐FU)‐based adjuvant therapy drugs are commonly used. 9Therefore, regarding new drug development, the therapeutic outcome of 5‐FU is often used as the baseline for comparison in patients with GCa.^7^ Studies have shown that 15% of patients with GCa are HER2 positive,^10^, ^11^ and such patients can be treated with a HER2 inhibitor as alternative once 5‐FU fails.

A study revealed that lipoprotein protein/receptor‐route‐mediated cholesterol import and the resulting steroidogenesis play crucial roles in GCa progression.^12^ CYP19A1 (cytochrome P450 family 19 subfamily A member 1; also named aromatase, Ar) is the key enzyme catalysing the conversion of androstenedione or testosterone to oestradiol or oestrone. Several studies have shown that breast cancer progression is also associated with Ar, whereas Ar inhibitors (ARIs) were also implemented in a therapeutic regimen.^1^, ^13^ Whether ARIs can be used for GCa therapy is an intriguing question. Several cohort studies have reported that Ar expression was higher in GCa tumours than it was in normal parts.^12^, ^14^, ^15^ This information has motivated the evaluation of the clinical value of Ar.

The mechanism of action (MOA) of ARIs involves inhibiting Ar‐enzymatic function^16^; this therefore reduces oestrogen levels in organisms.^17^, ^18^ ARIs are commonly used in the clinical treatment of breast cancer.^19^ They constitute an adjuvant hormonal therapy for patients with oestrogen receptor (ER) or for postmenopausal patients to reduce breast cancer risk.^20^ ARIs can be classified as type I (ARI‐I; eg, anastrozole and letrozole [nonsteroidal]) and type II (ARI‐II; eg exemestane [steroidal]).^18^, ^21^ However, these two types of ARIs act in different inhibitory modes against Ar ARI‐I interacts with the catalytic site of Ar by inhibiting anastrozole conversion. The mode of action of ARI‐I entails reversibly blocking the interaction between anastrozole and Ar, thus leading to elevated Ar levels in cells. By contrast, ARI‐II irreversibly inhibits Ar by forming a covalent bond with the catalytic site of Ar Thus, ARI‐II irreversibly inactivates Ar function, leading to diminished Ar levels in cells.^22^ Additionally, the different modes of action of ARI‐I and ARI‐II trigger diverse molecular mechanisms, although they target the same proteins.^23^

In this study, we used a bioinformatics approach to explore the suitability of Ar as a targeting agent for treating GCa, thus meeting clinical needs. We also tested the value of targeting Ar with ARIs in GCa preclinical models and provide new insight into ablating Ar with a non‐irreversible inhibitor in GCa treatment.

## 2 MATERIALS AND METHODS

### 2.1 Patients

This study was approved by the Ethics Committee of the Institutional Review Board of Kaohsiung Medical University Hospital (KMUH‐IRB‐20120176). Informed consent was obtained from all participants in accordance with the Declaration of Helsinki. Primary tumour tissues were obtained from 220 GCa patients undergoing surgical resection at Kaohsiung Medical University Hospital between 2007 and 2014. Patient characteristics and clinical outcome were followed until death, censorship or loss to follow‐up. Gastric tumour tissue cores were collected from each patient and used to construct a tissue microarray (TMA).^24^ The clinical parameters and overall survival (OS) data were obtained from patients’ medical records followed up for 5 years.

### 2.2 Kaplan‐Meier plotter for cancer survival analysis

To analyse the association of survival with gene expression, a web‐based Kaplan‐Meier plotter (http://kmplot.com/analysis/index.php?p=service&cancer=gastric) was used and a log‐rank test was used to assess the differences between patient groups stratified according to the median of gene expression. A *P*‐value of <.05 was considered statistically significant.

### 2.3 Statistical analysis

Statistical analyses were performed using Student's *t* test. All experiments were repeated at least three times, and *P*‐value <.05 was considered to be statistically significant.

Other materials and methods (including reagents, cytotoxic assay, IC.50 measurement, colony‐forming assay, gene expression measurements, knockdown of Ar in the cells and cancer cells xenograft assay) are in the online supplemental materials .

## 3 RESULTS

### 3.1 Ar expression is the GCa prognosis biomarker

**Figure 1:**
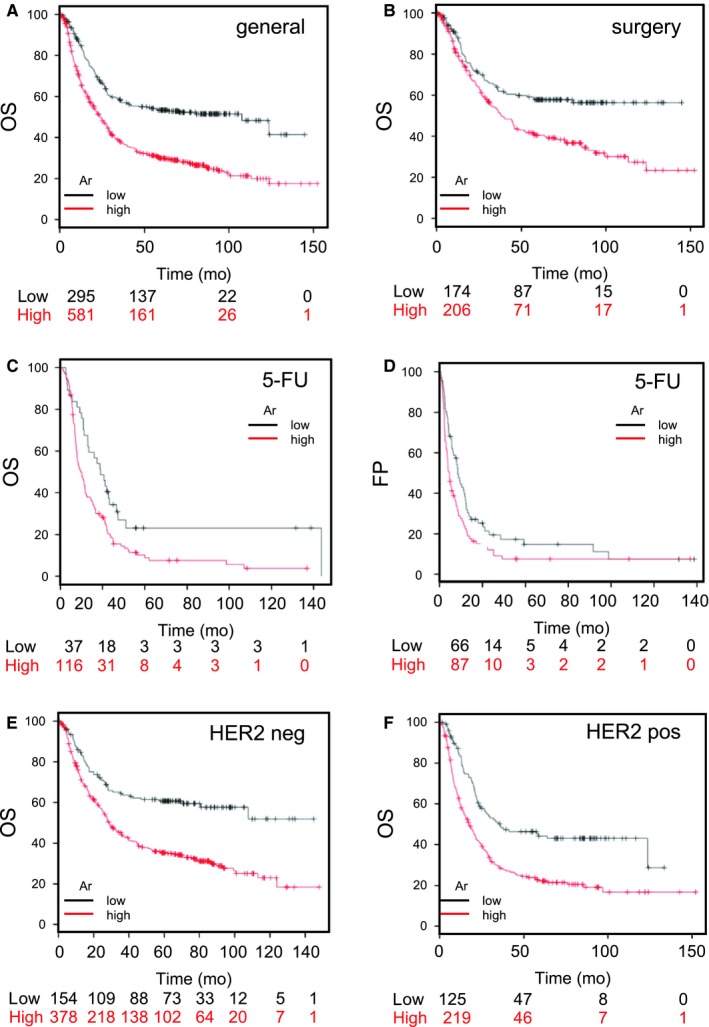
Ar expression is the gatekeeper of gastric cancer (GCa) prognosis. A, OS curve associated with Ar expression status in general patients with GCa. The red line indicates high expression, and black line indicates low expression. At the initial time‐point (0 mo), 581 patients had high Ar expression levels and 295 had low Ar expression levels. At the last time‐point (150 mo), one patient had high Ar expression and none of the patients had low Ar expression. The corresponding HR was 1.98, and P‐value was 3.6e‐12. B, OS curve associated with Ar expression status in GCa patients with surgery. The corresponding HR was 1.73, and P‐value was .00028. C, OS associated with Ar expression status in patients with GCa who underwent 5‐FU therapy. The corresponding HR was 2.22, and P‐value was 4.1e‐10. D, PFS (after therapy; 150 mo) after associated with Ar expression status in patients with GCa who underwent 5‐FU therapy. The corresponding HR was 1.51, and P‐value was .019. E, OS associated with Ar expression status in patients with GCa who were HER2 negative. The corresponding HR was 2.07 and P‐value was 2.6e‐07. F, OS associated with Ar expression status in patients with GCa who were HER2 positive. The corresponding HR was 1.94, and P‐value was 4.7e‐06

To assess the effect of Ar expression in clinical settings, we implemented two strategies to evaluate the roles of Ar in human GCa progression: one of the strategies entailed conducting a single‐hospital cohort TMA immunohistochemistry study on an Asian population (Tables 1 and 2); the other entailed using a Kaplan‐Meier plotter for survival analysis to determine the association of gene expression with Caucasian GCa prognosis (Figure 1). In the single‐cohort TMA study, men constituted a predominant portion of the study population (111 men vs 65 women). The Ar‐positive staining can be found in men (14%) and women (22%). The positivity in normal parental was higher in women (67%) than it was in men (41%) (Table 1; *P* = .0017). The GCa death rate was higher (*P* = .0283) in women (46%) than it was in men (27%). Notably, the association of tumour Ar positivity with death rate was higher in men (10 of 16 high‐expression patients died [63%], whereas 15 of 95 low‐expression patients died [24%], *P* = .0019). We observed comparable death rates between high (eight out of 14 patients died; 57%) and low (22 out of 51 patients died; 43%) Ar expressions among women. In normal parental lesions, the effect of Ar expression was significant (*P* = .0283) in women, where low expression was associated with a high death rate (16 out of 22 patients; 73%) and high expression was associated with a low death rate (13 of 44 patients; 30%). These data indicate a notable effect of Ar expression on sex, prognosis and microenvironmental regulations.

**Table 1:** Gastric cancer (GCa) cohort demograph and aromatase expressions

	Sex	Age of diagnosis		Ar score (in tumour)			Ar score (NP)	
	Number	mean ± SEM	*P*‐value*	High	Low	*P*‐value*	High	Low
Male	111 (63%)	63.2 ± 1.17	n.s.	16	95	.2253	46	63
				14%	86%		41%	57%
Female	65 (37%)	64.3 ± 1.71		14	51		44	22
				22%	78%		67%	33%

Abbreviation: NP, normal parental.

* The Pearson chi‐square *P*‐value comparing male vs female.

**Table 2:** Aromatase expression and gastric cancer (GCa) survival

	Death/rate%			Ar score (in tumour; death/survive; rate%)			Ar score (NP; death/survive; rate%)		
	Death	Survive	*P*‐value†	High	Low	*P*‐value‡	High	Low	*P*‐value‡
Male (n = 111)	33/27%	78/70%	.0283^†^	10/6	15/80	.0019*	15 (46)	18 (63)	.7401
				63%	24%		33%	29%	
Female (n = 65)	30/46%	35/55%		8/6	22/29	.351	13/ 31	16/ 6	.0011‡
				57%	43%		30%	73%	

Abbreviation: NP, normal parental.

* High expression: IHC score ≥2; low expression: IHC < 2.

^†^ The Pearson chi‐square *P*‐value comparing male vs female.

^‡^ The Pearson chi‐square *P*‐value comparing high vs low Ar expression.

Assessing a gene expression database in the Caucasian population revealed that high Ar expression was associated with poor prognosis (OS and free progression [FP]) in patients with GCa stratified according to multiple clinical classifications and therapy modalities. The positive association of Ar expression with treatment modalities implies that Ar has potential to serve as a targeting agent to fulfil unmet medical needs. Figure 1A‐F demonstrates the importance of Ar expression in patients’ disease progression in general, after surgery and after 5‐FU treatment, as well as in HER2 positivity. The hazard ratio (HR) was 1.98 for general OS, 1.73 for postsurgery OS, 2.22 for 5‐FU treatment OS, 1.51 for FP after 5‐FU treatment, 2.07 for OS in HER2‐negative patients and 1.94 for OS in HER2‐positive patients. The results show that high Ar expression was correlated with patient prognosis at different parameters in GCa, suggesting the potential of Ar as a new target for GCa.

### 3.2 Modulation of Ar expression suppresses GCa cell growth

**Figure 2:**
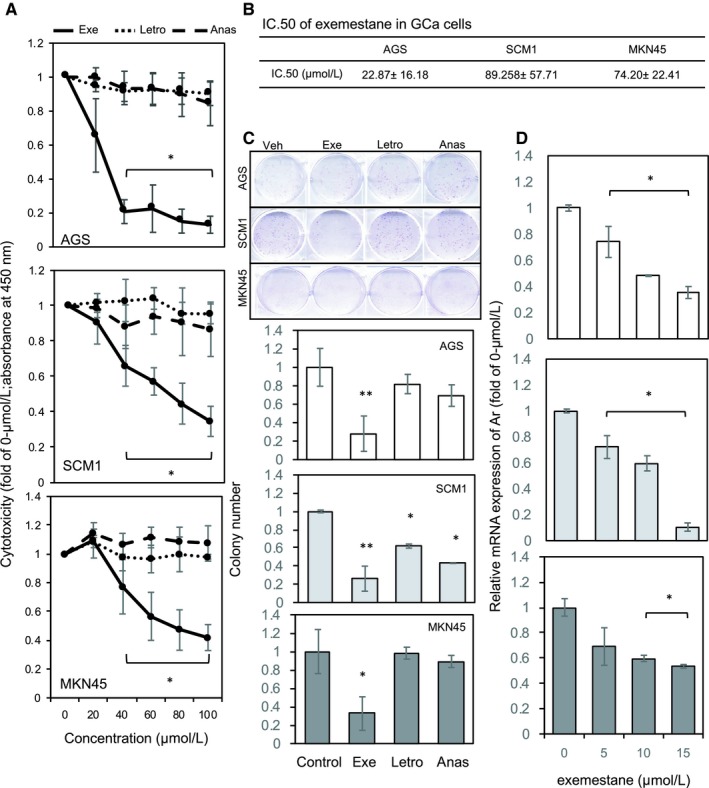
Differential cytotoxic effects of ARIs on gastric cancer (GCa) cells. A, The cytotoxic effect of ARIs was determined using WST‐1 cytotoxicity assay conducted on GCa cells (AGS, SCM‐1 and MKN45). The mean absorbance (450 nm) showed the viability of GCa cells treated with increasing concentrations (0, 20, 40, 60, 80 and 100 μmol/L) of various ARIs for 48 h (type I: anastrozole and letrozole; type II: exemestane). B, IC50 values indicating the cytotoxic efficacy of exemestane against GCa cells were calculated using CalcuSyn software. C, Cell growth suppression effect of ARIs was measured using a colony formation assay, and the results showed that the ARIs exhibited different efficacy levels in suppressing GCa cell growth. Long‐term (2 wk) and low‐dose (20 μmol/L) treatment showed various inhibitory efficacy levels. D, Down‐regulation of Ar by treating GCa cells with exemestane. GCa cells were treated with exemestane (0, 5, 10 and 15 μmol/L) for 48 h, and then, Ar mRNA was analysed using qRT‐PCR. *, ** and *** indicate significant differences with *P*‐values <.05, .01 and .001, respectively

As mentioned, two MOAs of ARIs were considered to test whether ARIs can be used for GCa therapy. Three ARIs (ARI‐I: anastrozole and letrozole, reversible inhibitor; and ARI‐II: exemestane, irreversible inhibitor) were introduced. Cytotoxicity (Figure 2A,2B) and colony formation capacity (Figure 2C) were measured, and the results revealed that exemestane had excellent cytotoxic against GCa cells. By contrast, we did not observe a significant cytotoxic effect of anastrozole or letrozole on GCa cells, implying that ARIs have a different mode of action in GCa. In addition, exemestane could suppress Ar expression at the transcriptional level (Figure 2D). The discrepancy in cytotoxic efficacy between ARI‐I and ARI‐II raised the question whether Ar expression but not enzymatic activity (concerting androgens to oestrogens) may be crucial for cytotoxic efficacy against GCa cells.

**Figure 3:**
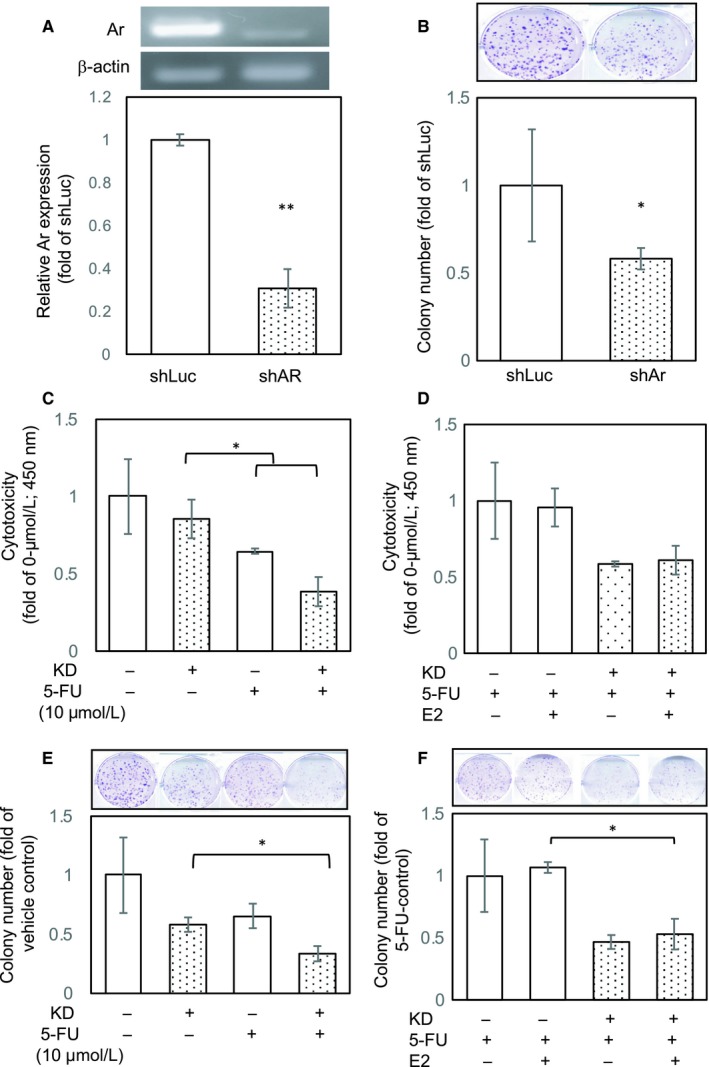
Expression, but not catalytic activity, of Ar affects gastric cancer (GCa) cell growth. A, Knockdown efficacy of Ar shRNA in AGS cells. Upper band densitometry results represent the PCR product of Ar cDNA, and lower bar chart represents the quantitation of RT‐PCR results. B, Cell growth of shAr compared with shLuc on AGS cells. The upper image shows the representative wells of the colony formation assay. The lower bar chart represents the quantitation result of the colony formation assay. C, Cytotoxicity of 5‐FU against shLuc and shAr AGS cells. D, Cytotoxicity of 5‐FU and/or oestradiol (E2; 10 nmol/L) against shLuc and shAr AGS cells. E, Colony‐forming ability of shLuc and shAr AGS cells treated with 5‐FU. F, Colony‐forming ability of shLuc and shAr AGS cells treated with 5‐FU and/or oestradiol (E2, 10 nmol/L)

To test this hypothesis, we introduced gene‐silencing technology with shRNA targeting Ar mRNA expression (Figure 3A). We compared the colony‐forming ability of shLuc cells with that of shAr infectants and found that the shAr infectants had a lower colony‐forming ability than did the shLUC infectants. To examine whether Ar down‐regulation can alter the sensitivity of 5‐FU to GCa, we tested the response of 5‐FU to shLuc and shAr infectants. The results are presented in Figure 3C, demonstrating an excellent synergistic effect in the combined treatment. To rule out the possibility of Ar‐enzymatic‐activity‐mediated oestradiol (E2) production, we added E2 to both shLuc and shAr infectants to examine whether E2 can prevent 5‐FU‐mediated cytotoxicity. The results demonstrated that E2 addition did not alter the result of combination treatment of Ar knockdown and 5‐FU (Figure 3D). In addition to the short‐term cytotoxic effect of the combined treatment, we observed that the combined treatment had a long‐term effect on colony formation in GCa cells. Specifically, as shown in Figure 3E,F, with a combination of Ar knockdown and 5‐FU, the colony‐forming ability was significantly reduced; adding E2 did not reverse the effect of the combined treatment, suggesting that 5‐FU cytotoxicity may be enhanced through Ar‐meditated signalling independent of oestrone conversion.

Overall, our findings provide proof‐of‐concept evidence that ablating Ar expression using exemestane or shRNA is potentially therapeutic.

### 3.3 Preclinical evaluation of 5‐FU and exemestane combination therapy

**Figure 4:**
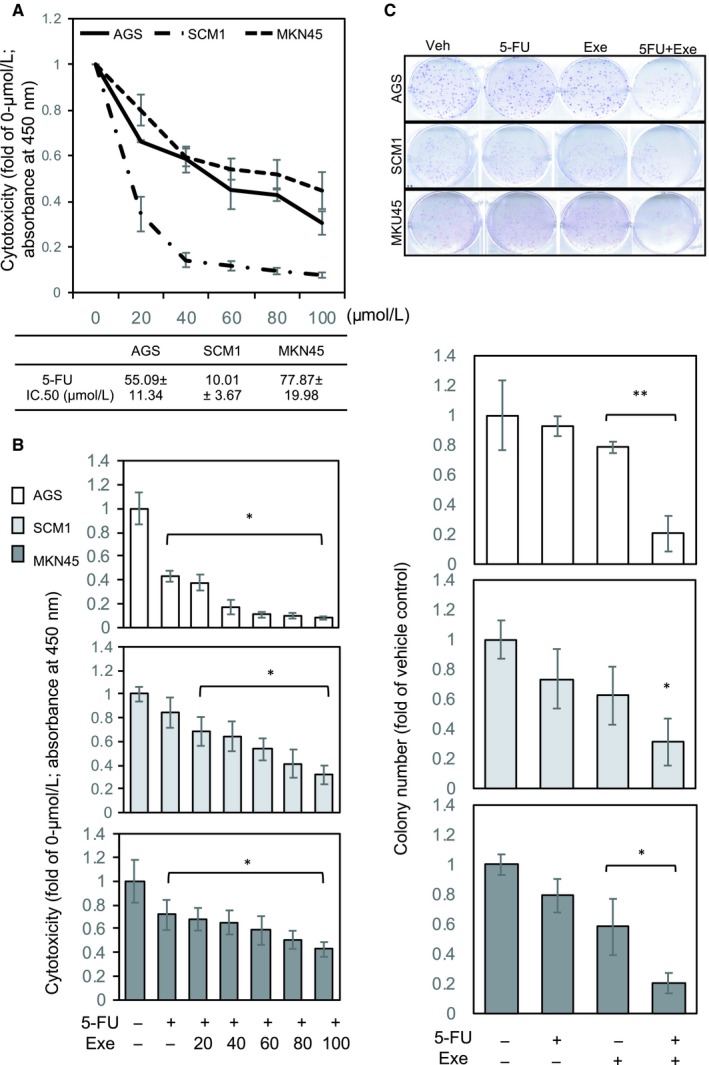
Combination treatment of exemestane and 5‐FU suppresses gastric cancer (GCa) cell growth in vitro. A, Cytotoxic efficacy of 5‐FU against GCa cells. Upper panel: mean absorbance (450 nm) was evaluated to determine the viability of GCa cells treated with 5‐FU (0, 20, 40, 60, 80, 100 μmol/L) for 48 h. Lower panel: 5‐FU cytotoxic IC50 values were calculated using CalcuSyn software. B, Cytotoxic efficacy of 5‐FU (10 μmol/L) and in combination with exemestane (20, 40, 60, 80 and 100 μmol/L) against GCa cells. C, Colony suppression efficacy of treatment with 5‐FU alone (10 μmol/L), treatment with exemestane alone (20 μmol/L) and combination treatment against GCa cells

As shown in Figure 3D,E, a synergistic inhibitory effect was observed for the combination treatment of Ar knockdown and 5‐FU. Combining exemestane and 5‐FU has high potential for GCa treatment. Therefore, we conducted a series of preclinical evaluations of the combination treatment in vitro and in vivo. First, we tested the cytotoxic efficacy of 5‐FU in three GCa cells (Figure 4A): AGS, MKN45 (less sensitive) and SCM1 (sensitive). The IC50 value observed for AGS was 55.9 ± 11.34, that observed for MKN45 was 77.89 ± 19.98, and that observed for SCM1 was 10.01 ± 3.67 (μmol/L). Subsequently, we measured the cytotoxic effect of the add‐on treatment with exemestane on the 5‐FU‐treated cells. The results revealed the synergistic (AGS cells) and additive (SCM1 and MKN45 cells) effects of the combination treatment (Figure 4B). Moreover, the combination treatment exhibited long‐term effects against colony‐forming ability (Figure 4C).

**Figure 5:**
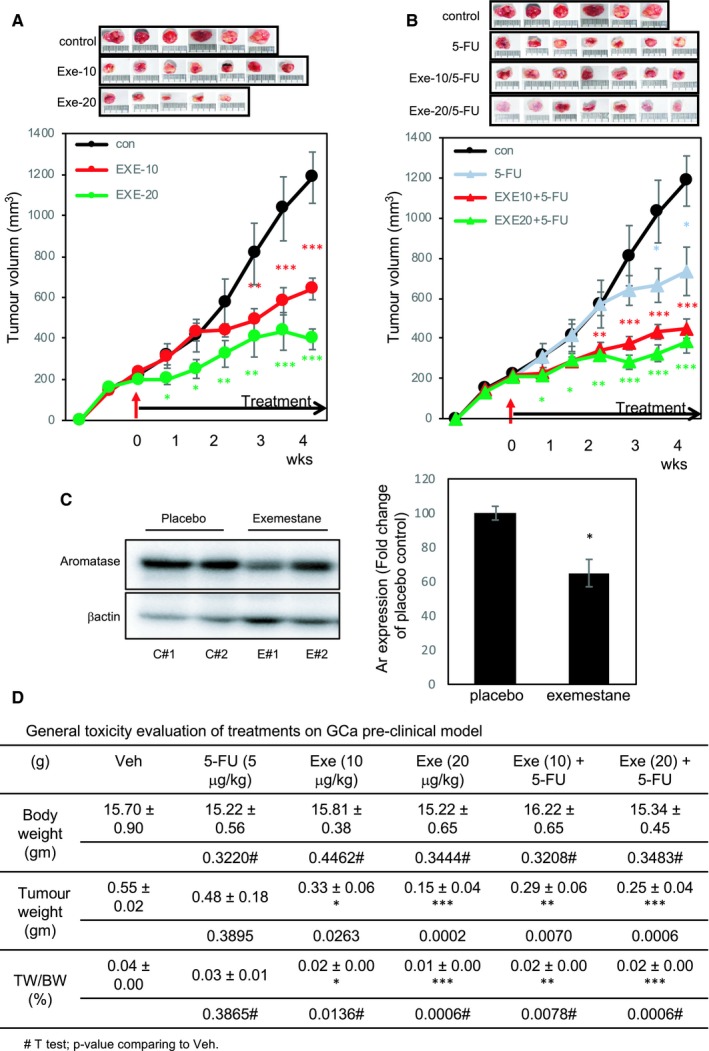
Combination treatment of exemestane and 5‐FU suppresses gastric cancer (GCa) tumour growth in vivo. A, MKN45 xenograft mouse model was used for testing tumour suppression effect of exemestane. The placebo (PBS, con, black line) or exemestane (low dose, 10 mg/kg, EXE‐10, red line; medium dose, 20 mg/kg, EXE‐20, green line) was intraperitoneally injected when the tumour size reached 200 mm^3^ three times per week for four consecutive weeks. The red arrow indicates the time of initial drug injection. B, The same GCa tumour model and treatment procedure were used to test the effect of combination treatment of exemestane and 5‐FU. Results for the placebo (PBS, con, black line), low‐dose 5‐FU (5‐FU, 5 mg/kg, grey line) or combination treatment of 5‐FU and exemestane (low dose, 10 mg/kg, EXE‐10, red line; medium dose, 20 mg/kg, EXE‐20, green line) are presented. C, Ar Immunoblot of xenografted tumour from placebo and exemestane (10 mg/kg) treated mice. Left‐handed side image is representative tumours, and the right‐handed side bar graft is the quantitation of five pairs of tumours. C#1 represented control group number 1 tumour; where E#1 represented exemestane # 1 tumour. D, Bodyweight, tumour weight and tumour weight‐to‐bodyweight ratio of GCa mice at the time of killing. # are the *P*‐value comparing groups with Veh using *t* test. *, ** and *** indicate significant differences for *P*‐values <.05, .01 and .001, respectively

With the success of the combination of exemestane and 5‐FU for treating GCa cells, we proceeded with subcutaneous implantation of MKN45 (5‐FU‐insensitive cells) in a xenograft mouse model of GCa in a 4‐week therapeutic term. As shown in Figure 5A, when the therapy started, the tumour size was 200 mm^3^. Subsequently, various drugs were intraperitoneally injected three times per week for four consecutive weeks. The tumour size decreased with exemestane treatment in a dose‐dependent manner (Figure 5A). A low dose (10 mg/kg/mouse) of exemestane could reduce the tumour size by approximately 50%, and a medium dose (20 mg/kg) of exemestane could reduce the tumour size by approximately 70%. Furthermore, a low dose (5 mg/kg) of 5‐FU slightly reduced the tumour size (65%), but add‐on treatment with exemestane could suppress the tumour size by approximately 90% (Figure 5B). Notably, the bodyweights were comparable among all groups. Tumour weight was significantly reduced in mice treated with exemestane (*P* = .0002), but mice receiving a low dose of 5‐FU alone did not show significant tumour growth inhibition (*P* = .3895). In addition, exemestane and 5‐FU could synergistically promote anticancer efficacy (from *P* = .0263 to *P* = .007). Considering the effects of the treatments on the general wellness of the experimental mice, we divided tumour weight by bodyweight (Figure 5C). We used it as the basis to compare within the treatment groups. We determined that combination treatment was the best scheme for therapy.

In sum, targeting Ar with exemestane (ARI‐II) might be a new effective therapeutic approach for GCa. Single therapy or combination usage with 5‐FU is worthwhile to be tested in clinical settings.

## 4 DISCUSSION

Gastric cancer is a complex malignancy because patients are usually diagnosed at an advanced stage and have a poor prognosis.^4^ Since the early 1980s, fluorouracil‐based chemotherapy is considered a standard treatment for GCa.^12^ However, 5‐FU is commonly associated with poor selectivity and systemic toxicity.^25^ Therefore, seeking a more effective target is crucial for GCa therapeutics. In this study, we first discovered that Ar expression might be a new prognostic biomarker and an important gatekeeper in multiple treatment modalities for patients with GCa; thus, Ar has potential to serve as a targeting agent to develop medications to fulfil unmet medical needs. Accordingly, targeting Ar seems to be a powerful strategy for drug development. This report provides proof‐of‐concept and preclinical evidence to support this concept. Several critical issues are discussed as follows.

### 4.1 Potential hazard of anti‐ER therapy for GCa treatment

The contribution of female factors to GCa development is controversial. For example, a large‐scale epidemiological survey indicated female factors, such as reproductive age, ovariectomy surgery, breastfeeding, pregnancy and contraceptive agents, were suggested that the oestrogenic signal suppresses GCa incidence.^26^ However, several study findings have contradicted this suggestion. For example, a large‐scale survey (1299 patients) indicated that female factors contribute to poor survival in GCa and that male factors contribute to GCa patient survival following surgery.^27^ The conflicting results between serum levels of sex hormones and receptor expression levels signify a possible role of intrinsic de novo synthesis of sex steroids in GCa. Recent studies using bioinformatics approaches to evaluate cholesterol importing, cholesterogenesis and steroidogenesis in patients with GCa^12^, ^28^, ^29^ have revealed that the possibility of circulating female hormones influencing GCa progression is small; nevertheless, endogenous steroidogenesis for female hormone production is the gatekeeping biochemical event in patients with GCa. Therefore, anti‐oestrogen or anti‐ERs have potential for application in GCa therapy. However, anti‐ERs (eg tamoxifen) may lead to the development of gastric, oesophageal and colorectal malignancies.^30^, ^31^, ^32^ In the current study, we targeted the upstream region of oestrogen/ER, oestrone synthesis, in cancer cells and demonstrated excellent tumour suppression efficacy.

### 4.2 Exemestane MOA of Ar expression suppression

Two types of ARIs are approved for treating cancer: ARI‐I, comprising reversible nonsteroidal inhibitors (eg anastrozole and letrozole), and ARI‐II, comprising irreversible steroidal inhibitors (eg exemestane and formestane). Both types of ARIs can inhibit oestrogen synthesis by targeting the catalytic binding site of Ar^33^ Although the ARI‐I–Ar interaction mode is unclear, several simulation studies have revealed that ARI‐I interacts with Ar by forming a hydrogen bond (hydrophilic) and hydrophobic interaction. They bind to haem iron and expel both ligands and oxygen from the enzyme.^33^, ^34^, ^35^ Notably, Ar expression was reported to be elevated by treatment with ARI‐I, suggesting the compensatory effects of oestrone production. However, exemestane binds to the substrate‐binding pocket of Ar and forms an irreversible covalent bond at Ar, leading to the induction of Ar degradation.^36^ Thus, the activity of ARI‐II prolongs the inhibitory effect of oestrogen synthesis depending on the Ar protein levels. In our study, we observed not only proteolytic degradation of Ar but also transcriptional inhibition of Ar The additional hydrophobic interactions through the C6‐methylidene group with a hydrophobic crevice been surrounded, which could add to exemestane binding affinity, thus providing better shape complementarity to Ar^37^ Regarding pharmacokinetics in humans, exemestane has a half‐life of approximately 24 hours,^38^ which is shorter than those of anastrozole (30‐60 hours) and Letrozole (42 hours). Exemestane first forms a reversible bond with Ki value 26 nmol/L in Ar and then converts into intermediate to inactive Ar through irreversible covalent interaction with a half‐life of 13.9 minutes. The long‐term degradation process of Ar was reported to be shortened, with a half‐life of 12 hours, compared with that of the control (half‐life = 28 hours).^39^ Exemestane can be rapidly absorbed with 42% oral bioavailability, and it reaches peak plasma concentrations within 2 hours following oral administration of a single 25‐mg dose. It also has high safety in humans, having no significant drug toxicity at doses of up to 600 mg/d, and it is well tolerated.^40^ Suggested by pharmaco‐toxicological studies of exemestane in human, we believe that introducing exemestane for down‐regulating Ar in GCa patients might be a feasible therapeutic approach.

### 4.3 Possible mechanism of Ar silencing with exemestane

Exemestane inhibits Ar expression, which is considered an essential MOA of GCa cell suppression. We propose several mechanisms of Ar silencing. The first mechanism is transcriptional regulation; for example, Foxl2 (forkhead box L2; a protein‐coding gene) binds to the sequence ACAAATA in the promoter region of the Ar gene through its forkhead domain. Foxl2 could also interact with the ligand‐binding domain of Ad4BP/SF‐1 (adrenal 4 binding protein/steroidogenic factor 1; a nuclear receptor essential for reproductive tissue development and endocrine regulation) through the forkhead domain to form a heterodimer and enhance Ad4BP/SF‐1‐mediated Ar transcription.^41^, ^42^ Furthermore, whether the inhibitory activity of ARI‐II against Ar suppresses Foxl2 activation requires further examination. The second mechanism is feedback inhibition; for example, the activities of Ar promoters (ie I.3, II and I.7) can be collaterally activated with the catalytic activity of Ar^43^ However, this hypothesis was not favoured in this study because ARI‐I did not exert suppressive effects on Ar expression (data not shown). The third mechanism is off‐target inhibition; for example, Ar activity could increase prostaglandin E2 (PGE2) binding to the G‐protein‐coupled PGE2 receptor to stimulate cyclic AMP production.^43^, ^44^ Whether the consequential PGE2 ablation with ARI‐II inhibits cell growth requires further examination. The fourth mechanism is the epigenetic modification of Ar by ARI‐II. For example, a potent Ar expression inhibition agent, namely LBH589, can selectively suppress the human Ar gene promoter I.3/II by reducing C/EBPδ levels. The decreased binding of C/EBPδ on Ar could increase the levels of acetyl‐histones on the promoter I.3/II, thus silencing Ar expressions.^45^ Another highly possible mechanism is that ARI‐II suppresses C/EBPδ to silence Ar transcription.

## 5 CONCLUSION

In this study, we observed that Ar is a crucial GCa prognostic biomarker. Suppressing Ar expression by using ARI‐II could be an excellent therapeutic strategy, particularly when ARI‐II is used in combination with 5‐FU. Additional pharmaceutical studies and human trials are encouraged.

## CONFLICT OF INTERESTS

There is no conflict of interests in this work.

## AUTHOR CONTRIBUTION

JC Yang and N Chang were responsible for the execution of experiments, pharmacological verification and drafting of the article. DC Wu, IC Wu and CJ Liu provided the clinical sample and performed statistical analyses. WC Cheng, WM Chung, FJ Lei, WC Chang and HC Lai assisted with immunoblot, IHC, statistical analyses, clinical consulting, compound validation, translational study design and article drafting. WL Ma initiated, supported and designed the study. HC Lai and WL Ma supported the study and finally approved the article.

## Data Availability

All the data in the work can be provided upon reasonable request.
