# Supplementary material for: Preclinical evaluation of exemestane as a novel chemotherapy for gastric cancer
Source: J Cell Mol Med. 2019 Sep 26;23(11):7417–26. doi: 10.1111/jcmm.14605 (PMC6815818; doi:10.1111/jcmm.14605)
Supplement: Supplementary file 1 [file JCMM-23-7417-s001.docx]

**Supplemental Materials**

**Chemicals and cell culture**

ARIs (exemestane, letrozole, anastrozole, 5-FU, and estradiol [E2] [E8875]) were purchased from Sigma-Aldrich (St. Louis, MO, USA). The human GCa cell lines AGS, SCM1, and MKN45 (purchased from the Food Industry Research and Development Institute in Taiwan [BCRC purchase number: 60210]) were cultured in RPMI 1640 medium (RPMI) (GIBCO) with 10% fetal calf serum and 1% penicillin/streptomycin (GIBCO). Immediatedly after purchased from BCRC, the cell number were expanded and cryo-preserved in liquid nitrogen upon usage. The experimental cell lines were maintained at 37°C in a humidified atmosphere of 5% CO_2_. And all the experimental cell lines will not be used over than 20 passages.

**Cytotoxic analysis and IC50 calculation**

The WST-1 assay (Roche, US) was used for assessing cell growth. Briefly, 2.5 × 10^3^ cells/well were seeded in 96-well plates with RPMI/10% fetal bovine serum (FBS) and were incubated for 24 hours. Drugs were then added, and the cells were incubated (single treatment: exemestane, letrozole, and anastrozole [20, 40, 60, 80, and 100 μM]; combination treatment: 5-FU (10 μM) + exemestane, letrozole, and anastrozole of various dosages [20, 40, 60, 80, and 100 μM]). After 48 hours of incubation, 1:10/v:v of WST-1 solution was added for 60-min incubation; subsequently, cell viability was measured through colorimetric detection with an ELISA plate reader (BECKMAN COULTER PARADIGM TM Detection Platform) at an absorbance of 450 nm to generate an optical density proportional to the relative abundance of live cells in the given wells. The values of 50% inhibition concentration (IC.50) [2] for each drug were determined using CalcuSyn software [3] (BioSoft).

**Colony formation assay**

A total of 500 cells/well were seeded in a 6-well plate and then treated (Vehicle, 5-FU: 10 μM, exemestane: 20 μM, combination) for 7 days. After treatment, 4% formaldehyde solution was used to fix the cells, and the cells were then immersed in crystal violet solution for 1 hour. Subsequently, crystal violet was washed off, and cell colonies were photographed. Colony numbers were quantitated using ImageJ (NIH; [https://imagej.net/ Welcome](https://imagej.net/%20Welcome)).

**Total RNA isolation, cDNA synthesis, and Quantitative real-time PCR analysis**

RNA extraction followed the procedures described in a previous study [4]. In each 100-mm dish, 1 × 10^6^ cells/well were seeded and incubated for 24 hours. After treatment for 48 hours, cells were lysed with 1 mL Trizol (Invtrogen), followed by a phenol/chloroform phase separation procedure, and then 2-propanol was added to precipitate RNA. Subsequently, RNA was rinsed, dried, and dissolved in RNase-free water. For first-strand cDNA synthesis, 5 μg of total RNA/sample was subjected to reverse transcription polymerase chain reaction (PCR) using the PrimeScript^TM^ RT reagent kit (TAKARA Bio Inc.).

A real-time detection system (Bio-Rad Laboratories, Inc., Hercules, CA, USA) and the KAPA^TM^ SYBR FAST One-Step qRT-PCR Kit (Kapa Biosystems, USA) were used according to the manufacturers’ instructions. Relative gene expression was determined by normalizing the expression levels of the target genes to the expression level of reference genes (actin). Threshold value (Ct) dynamics were used (2^-ΔΔCt^) for the quantitation of gene expression. The qRT-PCR primer sequences used were as follows: *Ar* forward, 5’-ACC CTT CTG CGT CGT GTC A-3’; *Ar* reverse, 5’-TCT GTG GAA ATC CTG CGT CTT-3’. The PCR products were loaded onto 2% agarose gel in 0.5× TAE containing 2.5 μL of HealthView Nucleic Acid Stain per milliliter (Genomics, Cat. NO.GN-NAS-100). After electrophoresis, the gel was photographed under ultraviolet light.

**Knockdown Ar with lentiviral-based shRNA in GCa cell**

In principle, the lentiviral production and infection procedures were performed as described in a previous study [5]. Cells were transfected with the following lentiviral plasmids: psPAX2 packaging plasmid and pMD2G envelope plasmid (Addgen, MA, USA). Lentiviral plasmid vectors containing shRNA genes targeting *Ar* (TRCN0000064315; shAr sequence: CCGGGCAACT ACTACAACCG GGTATCTCGA GATACCCGGT TGTAGTAGTT GCTTTTTG) and the control plasmid (pLKO.1-shLuciferase) (National RNAi Core Facility, Academia Sinica, Taiwan) were also used. The lentiviral plasmids were cotransfected with psPAX2 and pMD2G into HEK293T cells at a ratio of 3:2:4 by using lipofectamine 2000 (Invitrogen, CA, USA). After transfection for 4–6 hours, the culture medium was replaced with 10 mL of fresh Dulbecco's modified Eagle's medium/10% FBS, and dishes were incubated at 37°C for another 48 hours. The culture supernatants containing recombinant lentiviruses were harvested, and dead cells and debris were removed using a 0.45-µm filter. After the infected cells reached 50%–60% confluence, they were washed and then incubated with 5 mL of fresh supernatants for 24–48 hours. The cells were then incubated in fresh RPMI containing puromycin (1 μg/mL) to eliminate uninfected cells.

**Experimental animal and xenograft implantation GCa model**

Six-week-old male BALB/cAnN.Cg-Foxnl^nu^/CrlNarl mice (National Laboratory Animal Center, Taiwan) were housed in specific pathogen-free conditions (Laboratory Animal Center of China Medical University Hospital, Taiwan). MKN45 cells (1 × 10^7^) were subcutaneously injected into both sides of the low back flank of mice. When the tumor volume reached 200 mm^3^, mice were randomly assigned to four groups (intraperitoneal; vehicle, 5-FU 5 mg/kg, exemestane 10 mg/kg, and combination) (n = 6~10 mice per group). The treatment term was three times a week for four consecutive weeks. After the mice were sacrificed, their tumor volume and tumor weight were measured. Concurrently, tumor sizes were obtained.

References:

1. Burleson, K.M., et al., *Disaggregation and invasion of ovarian carcinoma ascites spheroids.* J Transl Med, 2006. **4**: p. 6.

2. Chou, T.C., *Drug combination studies and their synergy quantification using the Chou-Talalay method.* Cancer Res, 2010. **70**(2): p. 440-6.

3. Chou, T.C. and P. Talalay, *Quantitative analysis of dose-effect relationships: the combined effects of multiple drugs or enzyme inhibitors.* Adv Enzyme Regul, 1984. **22**: p. 27-55.

4. Chung, W.M., et al., *MicroRNA-21 promotes the ovarian teratocarcinoma PA1 cell line by sustaining cancer stem/progenitor populations in vitro.* Stem Cell Res Ther, 2013. **4**(4): p. 88.

5. Chung, W.M., et al., *Ligand-independent androgen receptors promote ovarian teratocarcinoma cell growth by stimulating self-renewal of cancer stem/progenitor cells.* Stem Cell Res, 2014. **13**(1): p. 24-35.
